# Characterization and Comparison of the Utilization of Facebook Groups Between Public Medical Professionals and Technical Communities to Facilitate Idea Sharing and Crowdsourcing During the COVID-19 Pandemic: Cross-sectional Observational Study

**DOI:** 10.2196/22983

**Published:** 2021-04-30

**Authors:** Helen Xun, Waverley He, Jonlin Chen, Scott Sylvester, Sheera F Lerman, Julie Caffrey

**Affiliations:** 1 Department of Plastic and Reconstructive Surgery The Johns Hopkins University School of Medicine Baltimore, MD United States; 2 Department of Psychiatry and Behavioral Sciences The Johns Hopkins University School of Medicine Baltimore, MD United States

**Keywords:** cognitive intelligence, communication, COVID-19, crowdsourcing, evidence-based, Facebook, Facebook groups, internet, social media, virtual communities

## Abstract

**Background:**

Strict social distancing measures owing to the COVID-19 pandemic have led people to rely more heavily on social media, such as Facebook groups, as a means of communication and information sharing. Multiple Facebook groups have been formed by medical professionals, laypeople, and engineering or technical groups to discuss current issues and possible solutions to the current medical crisis.

**Objective:**

This study aimed to characterize Facebook groups formed by laypersons, medical professionals, and technical professionals, with specific focus on information dissemination and requests for crowdsourcing.

**Methods:**

Facebook was queried for user-created groups with the keywords “COVID,” “Coronavirus,” and “SARS-CoV-2” at a single time point on March 31, 2020. The characteristics of each group were recorded, including language, privacy settings, security requirements to attain membership, and membership type. For each membership type, the group with the greatest number of members was selected, and in each of these groups, the top 100 posts were identified using Facebook’s algorithm. Each post was categorized and characterized (evidence-based, crowd-sourced, and whether the poster self-identified). STATA (version 13 SE, Stata Corp) was used for statistical analysis.

**Results:**

Our search yielded 257 COVID-19–related Facebook groups. Majority of the groups (n=229, 89%) were for laypersons, 26 (10%) were for medical professionals, and only 2 (1%) were for technical professionals. The number of members was significantly greater in medical groups (21,215, SD 35,040) than in layperson groups (7623, SD 19,480) (*P*<.01). Medical groups were significantly more likely to require security checks to attain membership (81% vs 43%; *P*<.001) and less likely to be public (3 vs 123; *P<*.001) than layperson groups. Medical groups had the highest user engagement, averaging 502 (SD 633) reactions (*P*<.01) and 224 (SD 311) comments (*P*<.01) per post. Medical professionals were more likely to use the Facebook groups for education and information sharing, including academic posts (*P*<.001), idea sharing (*P*=.003), resource sharing (*P*=.02) and professional opinions (*P*<.001), and requesting for crowdsourcing (*P*=.003). Layperson groups were more likely to share news (*P*<.001), humor and motivation (*P*<.001), and layperson opinions (*P*<.001). There was no significant difference in the number of evidence-based posts among the groups (*P*=.10).

**Conclusions:**

Medical professionals utilize Facebook groups as a forum to facilitate collective intelligence (CI) and are more likely to use Facebook groups for education and information sharing, including academic posts, idea sharing, resource sharing, and professional opinions, which highlights the power of social media to facilitate CI across geographic distances. Layperson groups were more likely to share news, humor, and motivation, which suggests the utilization of Facebook groups to provide comedic relief as a coping mechanism. Further investigations are necessary to study Facebook groups’ roles in facilitating CI, crowdsourcing, education, and community-building.

## Introduction

SARS-CoV-2, first discovered in Wuhan, China, on December 31, 2019, has quickly spread among >16 million individuals worldwide by June 2020 [1], and has resulted in the disruption of activities of daily life [2,3]. Social distancing has emerged as a method of reducing the transmission of COVID-19 and includes government-recommended or -mandated policies to “remain at home” or quarantine. Strict social distancing measures have led people to rely more heavily on social media as a means of communication and information sharing [4], including crowdsourcing (using resources from many people to obtain a final goal). Forums that facilitate discussion and sharing of ideas, such as Facebook groups, allows for the democratization of information and permits the development of quick collaborations to allow for the allocation of resources and advancement of science and technology. Social media has played a critical role in the COVID-19 pandemic, as multiple social media forums were developed by medical professionals, laypeople, and engineering or technical groups to discuss current issues and possible solutions to the current medical crisis.

However, despite the benefits of message sharing and crowdsourcing on social media platforms, studies have shown that social media platforms can lead to the propagation of misinformation [5]. With the rapid dissemination of information through unregulated forums, it is often difficult to distinguish evidence-based posts and forums from those that are not validated or originate from a credible source. For example, during the COVID-19 pandemic, there has been great debate on whether social media platforms have bred unnecessary fear and facilitated the spread of misinformation [6]. While social media is a powerful medium for communication, it can also result in conflicting information and negative societal impacts.

Consequently, it is critical to understand how social media can be used effectively, especially during unprecedented times such as the current COVID-19 pandemic. The CoV-IMPACT consortium has called for “the development of a real-time information sharing system, drawing from data and analyses from a range of social media platforms, in multiple languages and across the global diaspora” [7]. Furthermore, social media has been used by medical professionals and researchers to communicate and form virtual communities through groups. In this study, we aim to characterize Facebook groups formed by laypersons, medical professionals, and technical professionals, with specific focus on information dissemination and requests for crowdsourcing.

## Methods

### Recruitment

Facebook was queried for user-created Groups with the keywords “COVID,” “Coronavirus,” and “SARS-CoV-2” at a single time point on March 31, 2020. The characteristics of each group were recorded, including language (ie, English or non-English), privacy setting (ie, public or private), security requirement to attain membership, and membership type (ie, laypersons, medical professionals, or technical professionals). For each membership type, the group with the greatest number of members was selected, and in each of these groups, the top 100 posts were identified using Facebook’s algorithm. Each post was characterized by category and subcategory, whether it was evidence-based or crowdsourced, and whether the poster self-identified. The coding scheme for category and subcategory was developed independently by 3 investigators (Table 1). Metrics were also recorded for these groups (ie, number of members and posts, adjusted to time on Facebook) and for posts (number of comments and number of reactions). Posts with duplicated content were discarded to avoid oversaturation of the sample.

**Table 1:** Predetermined coding framework for post categories.

Subcategories			Example posts
**Education and information sharing**			
	News	“Official statement from Dr. Peter Tsai, inventor of the electrostatic charging technology that makes the filter media of face masks including medical and N95.”	
	Academic	“I created these quick sheets (PDF and images) for non-ICU clinicians (medical or surgical) who may find themselves taking care of critically ill patients.”	
	Question	“Has anyone seen patients whose presenting symptom was only abdominal pain (no diarrhea)?”	
	Personal experience	“We recently had a COVID patient with a cimino fistula with thrombosis of the fistula.”	
	Resource	“As you know, the CARES act passed a few days ago and it is 800 pages long. There are a lot of provisions in it that may help you, whether you run a small business or are an employee of a health care facility.”	
	Movement-based advocacy	“Stay Home Stay Safe.”	
**Supply and equipment**			
	Idea sharing	“Here is a link to a google doc on how to make one yourself.”	
	Request for resources (demand)	“We need a way to make more. Can you help produce these?”	
	Offer to provide resources (supply)	“I’m in Miami looking to donate some face shields locally does anyone here need?”	
	Networking	“Anyone here been in touch with the NHS… Any contacts appreciated.”	
**Opinions**			
	Professional	“To summarize, treat your patients as individuals. If they have compliant lungs but are hypoxemic, use PEEP cautiously, and if they are not PEEP responsive, don’t persist in trying to treat them for a disease they probably don’t have.”	
	Layperson	“I have remained fairly calm since January when the news broke, but today I find myself sad and weeping for all that the world has suffered.”	
	Conspiracy theory	“Russia and anti-vaxxers are spreading disinformation about COVID-19 and 5G.”	
**Humor and motivation**			
	Humor	“‘What’s parenting during lockdown like?”	
	Support for health care workers	“We love you guys....thank you for saving life in this hard time”	
	Inspiration	“Raise your hand if you know what it’s like to lose everything and rebuild your life from scratch.”	
	Mental health visibility	“For all the health care providers and unsung heroes on the front lines: nothing can “fix” these feelings, but maybe naming them and noticing them can make them a little easier to bear.”	

### Statistical Analysis

STATA (version 13 SE, Stata Corp) and Python (version 3.7.7, Python Software Foundation) were used for statistical analysis. Demographic data were tabulated and stratified by the type of group (medical, layperson, or technical). Hypothesis testing was conducted with a Cronbach *α* of .05. To compare membership volume across Facebook groups, a Mann–Whitney *U* test for nonparametric data was used. A Kruskal–Wallis test was used to compare the volume of reactions and comments for the top 100 posts across various types of Facebook groups. Planned posthypothesis testing was conducted using the Dunn test. Lastly, the chi-square test was conducted to compare evidence basis by group type for the top 100 posts.

## Results

### Group Characteristics

**Figure 1:**
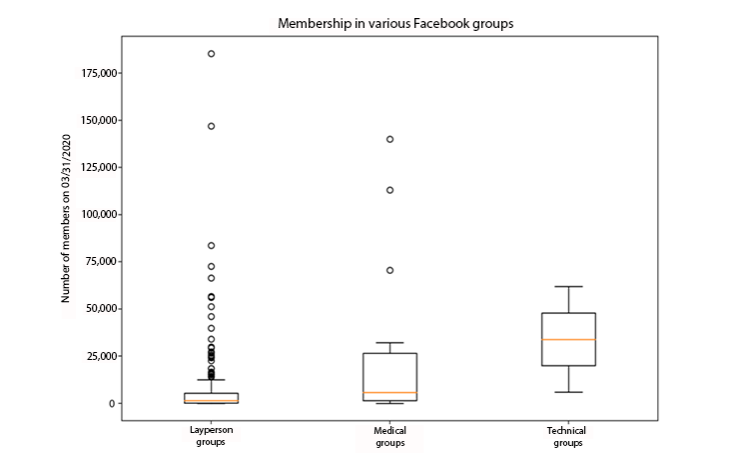
Characteristics of COVID-19–related Facebook groups by group type.

Our search on March 31, 2020, yielded 257 COVID-19–related Facebook groups (Table 2). Majority of the groups (n=229, 89%) were for laypersons, 26 (10%) were for medical professionals, and only 2 (1%) were for technical professionals. While the mean number of group members was 9203, groups ranged widely in size from 1 to 185,340 members. A Mann–Whitney *U* test indicated that overall, the number of members was significantly greater in medical groups (21,215, SD 35,040) than in layperson groups (7623, SD 19,480) (*P*<.01) (Figure 1 and Table 3). The mean number of group posts per day was 62 (range 0-625). Almost half of the groups were public (n=128, 50%); layperson groups (n=123, 54%) were more likely to be public than medical groups (n=3, 12%; *P*<.001). The majority of groups (n=218, 85%) predominantly operated in English, with no significant difference among layperson, medical, and technical groups (Figure 1).

**Table 2:** Characteristics of COVID-19–related Facebook groups (N=257).

Group characteristics			Value
**Type of group, n (%)**			
	Layperson	229 (89)	
	Medical	26 (10)	
	Technical	2 (1)	
Number of members, mean (range)			9203 (1-185,340)
Number of posts per day, mean (range)			62 (0-625)
**Privacy setting, n (%)**			
	Public	128 (50)	
	Private	129 (50)	
**Security requirement to join the group, n (%)**			
	Yes	121 (47)	
	Request	114 (44)	
	No	22 (9)	
**Language, n (%)**			
	English	218 (85)	
	Non-English	39 (15)	

**Table 3:** Characteristics of layperson, medical, and technical groups.

Characteristics			Groups				
			Layperson		Medical		Technical
Number of members, mean (SD)			7623 (19,480)^a^		21,215 (35,040)^a^		33,948 (39,578)
Public privacy setting, n (%)			123 (54)^b^		3 (12)^b^		2 (100)
**Security requirement to attain membership, n (%)**							
	Yes	98 (43)^c^		21 (81)^c^		2 (100)	
	Request	110 (48)		4 (15)		0	
	No	21 (9)		1 (4)		0	
English language, n (%)			193 (84)		20 (77)		2 (100)

^a^Significant difference in the number of members between layperson and medical groups (*P*<.01).

^b^Significant difference in public privacy setting between layperson and medical groups (*P*<.001).

^c^Significant difference in the presence of security requirements to attain membership between layperson and medical groups (*P*<.001).

Nearly all groups required prospective members to submit a request or to answer security questions to attain membership (n=235, 91%) (Figure 1). Medical groups were significantly more likely to require security checks to attain membership (ie, providing practice numbers, identification, verification of physicians, and agreement to the terms of the group) than layperson groups (81% vs 43%; *P*<.001). Among medical groups, the majority were private groups (n=23, 88%) that enforced security settings, with 4 groups (15%) that required requests, and only 1 (4%) that had no security settings. Similarly, both technical groups required security requirements to attain membership (n=2, 100%). In contrast, 98 (43%) layperson groups had security requirements, 110 (48%) had requests to join, and 21 (9%) had no security requirements.

**Figure 2:**
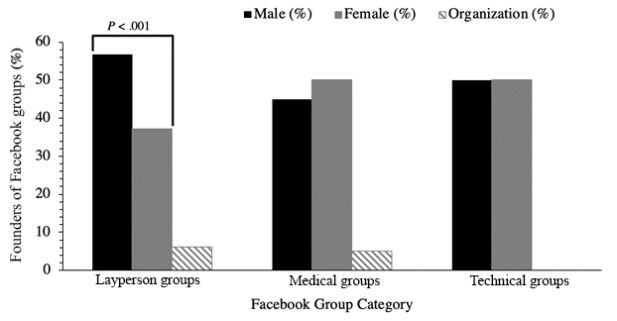
Gender of the creator of COVID-19–related Facebook groups by group type.

When investigating the gender of the Facebook group creator (male, female, or organization; Figure 2 and Table 4), layperson groups were more likely to be created by a male (n=131, 56.7%) rather than a female creator (n=86, 37.2%) (*P*<.001). Male creators were more common in non-English layperson groups than female creators (66.7% vs 28.2%; *P*<.001). Medical and technical groups were equally likely to be formed by a male or female creator. Facebook groups formed by organizations accounted for 14 (6.1%) layperson groups, 1 (5%) medical group, and no technical group.

**Table 4:** Distribution of creator genders (male, female, or organization) among layperson, medical, and technical groups.

Groups		Creator gender		
		Male, n (%)	Female, n (%)	Organization, n (%)
**Layperson**		131 (56.7)	86 (37.2)	14 (6.1)
	Predominantly English language	105 (54.7)	75 (39.1)	12 (6.3)
	Predominantly non–English language	26 (66.7)	11 (28.2)	12 (6.3)
**Medical**		9 (45.0)	10 (50.0)	1 (5.0)
**Technical**		1 (50.0)	1 (50.0)	0 (0.0)

### Post Characteristics

The largest layperson group (CoronaVirus International) was formed in late January 2020, while the largest medical (COVID-19 USA Physician/APP Group) and technical (Open Source COVID19 Medical Supplies) groups were formed in mid-March 2020. As of this writing, CoronaVirus International had 185,340 members, averaged at 333 posts per day, operated predominantly in English, and required answering security questions to attain membership. COVID-19 USA Physician/APP Group had 140,018 members, averaged at 100 posts per day, operated predominantly in English, and required answering security questions to attain membership. Open Source COVID19 Medical Supplies had 61,935 members, operated predominantly in English, and required security questions to attain membership.

**Figure 3:**
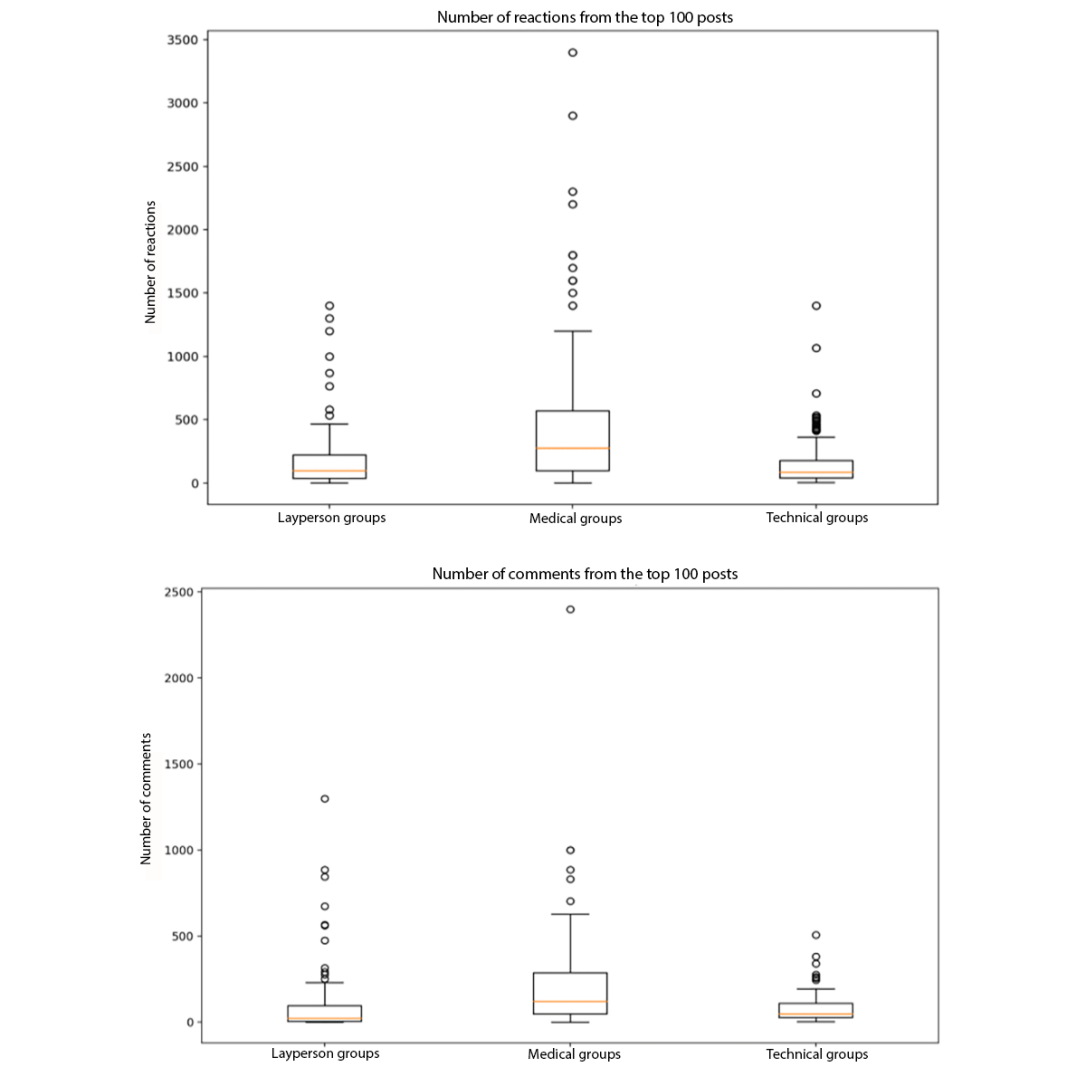
Average number of reactions and comments among the top 100 posts of COVID-19–related Facebook groups by group type.

Medical groups had higher user engagement, averaging at 502 (SD 633) reactions (*P*<.01) and 224 (SD 311) comments (*P*<.01) per post than layperson (182, SD 265 reactions and 104, SD 207 comments per post) and technical (165, SD 216 reactions and 80, SD 86 comments per post) groups (Figure 3 and Table 5).

**Table 5:** Member engagement in layperson, medical, and technical groups.

Engagement types	Groups				*P* value^a^
	Layperson	Medical	Technical		
Number of reactions, mean (SD)	182 (265)	502 (633)	165 (216)	<.01	
Number of comments, mean (SD)	104 (207)	224 (311)	80 (86)	<.01	

^a^*P* values for comparisons between layperson and medical groups.

On comparing the characteristics of the posts by groups (Table 6), layperson and medical groups had predominantly education and information sharing posts. Layperson groups had more posts that shared news about COVID-19 (31 vs 10; *P*<.001), while medical groups had more evidence-based posts (21 vs 4; *P*<.001). Technical groups predominantly contained posts related to supply and equipment sharing (n=99) compared to layperson and medical groups (*P*<.001), the majority sharing ideas (n=72), followed by posts related to networking (n=11), requests for resources (n=9), and offers to provide resources (n=7). Medical groups had more posts related to supply and equipment than layperson groups (17 vs 3; *P*<.001), including posts sharing ideas (11 vs 1; *P*<.001). Medical groups provided more professional rather than layperson opinions (18 vs 0; *P*<.001), while layperson groups provided more layperson opinions (12 vs 1; *P*<.001). For each layperson or medical professional group, there was only one post. Layperson groups were more likely to share posts related to humor and motivation than medical groups (29 vs 3; *P*<.001), including humor (21 vs 0; *P*<.001) and inspiration (4 vs 0; *P*<.001).

Medical group posters were significantly more likely to self-identify (91 vs 1; *P*<.001), and more likely to request crowdsourcing in the group (38 vs 19; *P*<.001) than their counterparts in layperson groups. There was no significant difference in the number of evidence-based posts among the 3 group types, with 28 evidence-based posts in layperson groups, 39 in medical groups, and 42 in technical groups (*P*=.10).

**Table 6:** Comparison of the characteristics of the top 100 posts by group type.

Types of posts				Groups						*P* value^a^	
				Layperson		Medical		Technical			
**Categories, n**											
	**Education and information sharing**		55		60		1		.48		
		News	31		10		0		<.001		
		Academic	4		21		0		<.001		
		Question	11		14		1		.52		
		Personal experience	5		7		0		.55		
		Resource	1		8		0		.02		
		Movement-based advocacy	3		0		0		.08		
	**Supply and equipment**		3		17		99^b^		<.001		
		Idea sharing	1		11		72		.003		
		Request for resources (demand)	2		2		9		.99		
		Offer to provide resources (supply)	0		1		7		.32		
		Networking	0		2		11		.16		
	**Opinion**		13		20		0		.18		
		Professional	0		18		0		<.001		
		Layperson	12		1		0		.002		
		Conspiracy theory	1		1		0		.99		
	**Humor and motivation**		29		3		0		<.001		
		Humor	21		0		0		<.001		
		Support for health care workers	4		2		0		.41		
		Inspiration	4		0		0		.04		
		Mental health visibility	0		1		0		.32		
**Evidence-based, n**											.10
	Yes		28		39		42				
	No		72		61		58				
**Crowdsourced, n**											.003
	Yes		19		38		42				
	No		81		62		58				
**Poster self-identified, n**											<.001
	Yes		1		91		12				
	No		99		9		88				

^a^*P* values for differences between layperson and medical groups.

^b^*P*<.001 on comparing between layperson and medical groups.

## Discussion

### Background

Information sharing on social media has become mainstream during the COVID-19 pandemic. In a matter of weeks, over 257 new groups were formed on Facebook, including those formed by laypersons, medical professionals, and technical professionals. In this study, we characterize how Facebook group activities surrounding the COVID-19 pandemic differ among layperson, medical, and technical groups, including members, user engagement, and types of posts.

### Principal Findings

Medical groups are more likely to be private, and require security questions and agreement with group policies, and posters were more likely to self-identify (providing details including their name, specialty, and location of practice) in accordance with the community rules, which is suggestive of a more professional community compared to layperson groups. Despite the heavier security, medical groups on average had more members than layperson groups (Figure 1 and Table 3) and higher engagement, with a larger number of reactions and comments per post (Figure 3 and Table 5). Strikingly, when characterizing the top 100 posts by group type, medical professionals were more likely to use Facebook groups for education and information sharing, including academic posts (*P*<.001), posts sharing ideas (*P*=.003), posts sharing resources (*P*=.02), and professional opinions (*P*<.001). Medical professionals were also more likely to request crowdsourcing than laypersons, asking questions about patient management and resources such as personal protective equipment. Together, this evidence suggests that medical professionals intentionally utilize Facebook groups as a forum to facilitate collective intelligence (CI) to compensate for the dynamic and unfamiliar evidence and guidance surrounding COVID-19 and associated treatments. CI is the “wisdom of crowds” [8], which refers to collective insight obtained from these groups [9-11], and has the potential to generate more accurate information or medical decision-making than individuals [12-14]. While previous studies on CI in medicine include activities such as case conferences and tumor boards [8], social media has been proposed as a facilitator of health information sharing [15] and CI across geographic distances [16]. Our findings highlight the power of social media to facilitate CI not only beyond geographic distances but also across additional physical barriers of strict social distancing practices owing to the COVID-19 pandemic, and intellectual barriers where conventional avenues of information searching and consulting are not yet available. Further studies are necessary to investigate whether participation in Facebook groups improves the knowledge base of medical professional participants and whether Facebook group CI influences decision-making.

However, layperson groups were more likely to share news (*P*<.001), humor and motivation (*P*<.001), and layperson opinions (*P*<.001) than medical groups. Layperson groups were less likely to crowdsource, and only 3% of posts were related to movement-based advocacy (such as “#stayathome”). This suggests that laypersons utilize the Facebook groups to form a community to share emerging news and share humor and inspiration, potentially to provide comedic relief as a coping mechanism. The COVID-19 pandemic has resulted in drastic shifts in day-to-day living for many individuals, including measures such as working from home, social isolation, adoption of hand hygiene, and wearing masks. These changes were rapid and may result in anxiety and distress among laypersons. Humor has been well evidenced as an adaptive mechanism for stress [17] and to reduce anxiety [18,19], enhance mood [19], and as a potential tool for psychotherapy [20-25]. The role of news sharing and providing humor and inspiration is analogous to that of a virtual support group, with the potential to connect individuals and foster reflections and conversations [26,27]. Consequently, it may be important for health care professionals to utilize these layperson Facebook groups to communicate with and educate laypeople and to understand their perspectives and experiences during the COVID-19 pandemic, provide supporting resources, and potentially facilitate grassroot movements (such as “#stayathome” and “#wearamask”).

The technical groups assessed in this study are a unique example of using Facebook groups for crowdsourcing, idea sharing, and networking worldwide. In total, 99 of the 100 top posts in the technical group analyzed herein were in regard to supply and equipment, 72 of which were related to idea sharing, such as open-sourcing designs for personal protective equipment, progress in designs for ventilators and ventilator splitters, etc. These groups had more evidence-based and crowdsourced posts than medical and layperson groups. The technical group serves as an example of the benefits and new standard of using Facebook groups for crowdsourcing and CI to cope with challenging times.

Layperson groups were significantly more likely to be formed by males. Surprisingly, this was not the case in medical and technical groups, where the group creators displayed an equal male:female gender distribution. This suggests that despite gender disparities in social media leadership positions globally, this gender gap is not evident in social media usage among medical professionals. Previous studies have reported that women in medicine in particular turn to social media for mentorship and networking [28,29] and that social media is a potential gender equalizer in medicine [30]. However, this does not discount persistent biases that may persist in Facebook group interactions. Consequently, additional studies are required to investigate how social media interactions occur and influence gender roles in medicine.

### Comparison With Prior Studies

Recent studies evaluating the utility of information sharing on social media have focused on negative effects including rapid dissemination of false information [5,7,9,10]. Misinformation propagated by social media is not unique to the COVID-19 pandemic. Previous studies have reported that only 53% of health-related posts by medical professionals on Twitter are supported by medical evidence [10]. Additionally, studies of social media posts related to the Ebola pandemic in 2014 reported a similar rate of false information [9,11]. Our study similarly reveals a small fraction of posts that are evidence-based, with an equal likelihood of a layperson’s post versus a medical professional’s post to be evidence-based (28 vs 39; *P*=0.10). Only 1% of posts from both medical and layperson groups were conspiracy theories, suggesting that a potential paucity of information surrounding the COVID-19 pandemic may explain the low number of evidence-based posts. Regardless, the potential to rapidly propagate misinformation on social media could be dangerous, in both medical professional and layperson groups. Layperson groups in particular, may benefit from a moderator or peer “champions” [31] to encourage evidence-based discussions and respectful user engagement.

Previous studies have also described the potential of Facebook groups as support groups and for community-building among patients [31-33], or the medical community as an educational tool to facilitate discussion, community-building, material sharing [34], and mentorship [35]. Our study complements this body of literature and highlights that virtual community-building on Facebook groups is accelerated during unprecedented times, such as the current COVID-19 pandemic. However, future studies are required to understand virtual community interactions and recommendations for the formation of impactful and secure Facebook groups.

### Limitations

A potential limitation to our study is the assumption that our findings are representative of the global culture of Facebook groups or other social media forums. We recognize that our study merely involves a small sample from among immensely diverse Facebook groups and the different communities that contribute to each group and the resulting culture. Furthermore, as a cross-sectional study, our data represent only 1 time point of the dynamic content on the social media platform. Our findings serve as a beachhead to establish the importance of understanding social media responses to the COVID-19 pandemic and its potential to facilitate CI, crowdsourcing, and community-building.

### Conclusions

In this study, we characterize how Facebook group activities surrounding the COVID-19 pandemic differ among layperson, medical, and technical groups. Medical professionals utilize Facebook groups as a forum to facilitate CI and are more likely to use the Facebook groups for education and information sharing, including academic posts, idea sharing, resource sharing, and professional opinions. Our findings highlight the power of social media to facilitate CI not only beyond geographic distances but also across additional physical barriers of strict social distancing practices resulting from the COVID-19 pandemic. Layperson groups were more likely to share news, humor, and motivation, which suggests the utilization of Facebook groups to provide comedic relief as a coping mechanism. Further studies are required to study the role of Facebook groups in facilitating CI, crowdsourcing, education, and community-building.

## Abbreviations

CI: collective intelligence
